# School stakeholders’ perceptions of physical education teachers’ core tasks—defining professional boundaries and increasing the relevancy of the subject

**DOI:** 10.3389/fspor.2026.1713275

**Published:** 2026-02-03

**Authors:** Sharon Tsuk, Ronnie Lidor, Michal Arnon, Matan Narkis

**Affiliations:** The Levinsky-Wingate Academic College, Netanya, Israel

**Keywords:** curriculum, health, promotion, relevance, sport pedagogy, task classification

## Abstract

**Introduction:**

Understanding how different groups of relevant school stakeholders perceive the scope of physical education (PE) and its impact on children and youth can assist policymakers and teachers to increase PE's relevancy at the current time.

**Methods:**

In the current study, we explored the perceptions of 381 school stakeholders of one main pillar of PE—the core tasks of the PE teacher in elementary and high schools. Five groups of school stakeholders were identified: PE college students (future PE teachers), PE teachers, coaches, sport and physical activity policymakers, and parents of children in elementary and high schools. The stakeholders were given a questionnaire where a list of potential tasks of the PE teachers was introduced. They were then asked to select up to five most important tasks for (a) PE teachers who worked at elementary schools, and (b) PE teachers who worked at high schools.

**Results:**

The most important core tasks of the PE teacher as perceived by the school stakeholders across the groups were (a) encouraging a healthy and active lifestyle, (b) promoting engagement in physical activity, (c) teaching a variety of sport disciplines, (d) enhancing children's self-confidence, and (e) developing sport-specific skills.

**Conclusion:**

Overall, the school stakeholders perceived the PE teacher as a health promoter for involvement in physical activity. These perceptions should be considered by policymakers who aim to increase the relevance of PE to school children's everyday lives.

## 1 Introduction

The quality and effectiveness of any subject (e.g., physical education; PE) taught in the school system (elementary, junior-high, and high schools) are largely shaped by the instructional/pedagogical tasks of the teacher (e.g., the PE teacher), whose influence extends beyond instruction to motivation, inclusion, and personal development (1–5). The main tasks of the PE teacher discussed in the literature include (a) providing physical activity (PA) time within a class period, (b) teaching skills and activities that transfer into PA outside of the PE class, and (c) encouraging students to be physically active (6–8). These tasks of the PE teacher are also endorsed in the PE program for elementary and high schools in Israel as proposed by the Ministry of Education (see: https://pop.education.gov.il/tchumey_daat/physical-education/elementary/physical-education-pedagogy/curriculum/).

The need to implement the aforementioned PE teacher tasks in school settings reflects what has already been established, namely that regular PA during childhood and adolescence is strongly associated with numerous physical, mental, and social benefits (9). PA contributes to healthy growth and development, supports cardiovascular and musculoskeletal health, and reduces the risk of chronic diseases (e.g., obesity and metabolic diseases) later in life (1, 10). Additionally, PA has been linked to improved mood, enhanced self-esteem, better concentration, and reduced symptoms of anxiety and depression among school-aged children (11–13). Moreover, participation in PA during school years is a key predictor of maintaining an active lifestyle in adulthood (14).

Despite the widespread recognition of the importance of PE and its contribution to the development of children and youth (5, 8), it is often perceived with low regard and considered less important than other academic subjects taught in schools, including language studies, mathematics, and science (15, 16). This may be due to a limited understanding of how different key PE-relevant stakeholders such as administrators, parents, policymakers, students, and teachers value the tasks of the PE teacher in practice [(6); see also (17)]. Understanding how relevant school stakeholders perceive the tasks of PE is important for two main reasons. First, their perceptions have the potential to impact several aspects of PE, such as the public and financial support it receives, the length/contents of PE teacher preparation, and the shaping of educational policy. Secondly, understanding the school stakeholders’ perceptions can assist both policymakers and teachers to increase the relevancy of PE at the current time. More specifically, teachers may, for example, be able to create better relevant learning personalized environments where every student is able to meet his/her own developmental needs. Relevant and personalized learning settings may contribute not only to the students’ own development, but also to their families and the local community since both serve as integral partners of the learning process (4). The more relevant the subject (PE) is, the more support it receives. This support may be given at two structural levels: the macro level—the national system (e.g., the Ministry of Education, Ministry of Health), and the micro level—the local/regional administration/authority and the school.

Several studies have examined stakeholders’ perceptions of different aspects of PE [see, for example, (6, 15, 18–20)]. Among the stakeholders who participated in these studies were administrators, school students, teachers, and parents. In one study on stakeholders’ perceptions, Fan and McMullen (6) explored the perceptions of 28 school students, classrooms teachers, administrators, and parents on the purpose and impact of PE in the western part of the United States. The participants were interviewed using a paired/group protocol. The findings of this study indicated that stakeholders’ understanding of the purpose of PE is closely tied to the concept of health. The stakeholders believed that in appropriately designed PE classes, students acquire knowledge about the importance of PA to healthy lifestyle, as well as practice activities that can be performed regularly outside the class/school. However, the stakeholders could not assess the actual impact of these PE activities on the students.

A qualitative study conducted in New Zealand (18) examined primary school classroom teachers’ perceptions of the value of PE. Data were collected in four primary schools and among eight classroom teachers. Using a thematic analysis and a constant comparative method, six subthemes under the postulated value of PE were identified: PE has benefits for physical well-being and health, the students learn social skills, PE shapes good character and self-efficacy, PE is for fun, good character and self-efficacy can bring into classroom learning, and PE is perceived as “a reset button”.

Another study, which included 127 school students and 68 PE teachers from 63 elementary schools in Indonesia (20), examined students’ and teachers’ perceptions of PE and teaching challenges. Data analyses showed that most students (95.6%) reported liking sport and PE. They preferred to learn PE through games, and they believed learning the subject had brought many benefits such as having a stronger and healthier body and increasing their motivation to be an outstanding athlete. However, most teachers (70.58%) felt that they were limited by low capacity to create engaging learning experiences and manage students in the learning processes.

Two main observations can be made based on the analyses of the stakeholders’ perceptions examined in the aforementioned studies: First, PE is viewed as an essential subject for the development of students in both elementary and high schools. Stakeholders believe that PE should be part of every school's curriculum in order to provide learners with positive experiences of PA. Second, although the stakeholders valued the importance of PE, they argued that they had difficulties to assess its impact on children and youth. Interestingly, these observations reflect perceptions of different groups of stakeholders from different international educational systems.

In Israel PE has been a core subject in the school curriculum—elementary, junior-high, and high schools. According to the regulations of the Ministry of Education (21), PE is taught twice a week, starting in the first year (Grade 1) of elementary school and ending in the last year (Grade 12) of high school. The two-weekly PE classes are scheduled on two different days in order to enable the students to be active at least twice a week. Each PE class lasts 45–50 min. In elementary schools, classes are usually taught for girls and boys combined. In junior-high and high schools, PE classes are taught separately for girls and boys in order to provide equal learning opportunities for both genders.

From an historical perspective, educational policymakers in Israel have adopted the PE definition proposed by the United Nations Educational, Scientific, and Cultural Organization [(22); see also (6)]. According to this definition, PE is a planned, progressive, active, inclusive, and peer-led learning-designed subject for children in kindergarten, elementary, and secondary schools, establishing the foundations for lifelong engagement in PA and sport. Given the significant amount of time children spend in school, PE classes are uniquely positioned to provide structured, developmentally appropriate opportunities for movement, motor skill acquisition, and the promotion of lifelong health behaviors.

In order to achieve the aforementioned goal of PE classes in Israel, PE students are required to complete a four-year preparation program, which is composed of three pillars: Pillar 1 consists of courses related to exercise and sport sciences, including applied anatomy, biomechanics, exercise physiology, health education, motor learning, philosophy of sport, and sport pedagogy. Pillar 2 consists of practical courses focused on the acquisition of motor skills associated with PA, sport, and leisure/recreational activities, such as basketball, folk dances, and volleyball. Lastly, Pillar 3 consists of teaching activities undertaken in the program, as well as in the field (schools). Upon meeting the academic requirements of the program, students receive a Bachelor of Education (BEd) degree and a teaching certification that allows them to teach PE in elementary schools, junior high schools, and high schools.

As with other educational systems, in Israel too, there is limited data on how school stakeholders value the importance of PE and its contribution to the development of children and youth. In one study (15), however, the perceptions of 150 school students, PE teachers, and school administrators regarding PE value, participation, and instructional support were examined by a cross-sectional survey. The results revealed that 83% of the students rated PE as equally important as other academic subjects taught in school, although about 70% of them reported that they received only one PE hour on a weekly basis. Students also reported lower environmental support for PE than teachers and administrators. In addition, PE teachers were observed as encouraging in class, although their influence outside school was less evident.

As emerged from the findings of the aforementioned studies [e.g., (6, 15, 18, 20)], different groups of stakeholders in various international educational systems perceived differently the impact of PE on students. In order to further explore how **school** stakeholders perceive PE, in the current study we aimed to focus on one aspect of the PE teacher—the expected of his/her core tasks in elementary and high schools. **By strengthening our understanding of what is expected of the PE teacher to do in class, we hope to also strengthen our understanding of what we have to do in order to increase the relevancy of PE at the current time.** Additionally, by identifying the consensus and divergence across tasks’ perceptions of relevant school stakeholder groups, we attempt to propose an evidence-based PE policy, as well as to continue the discussion on the best ways to establish more coherent, inclusive, and pedagogically sound approaches to PE in schools.

An evidence-based discussion on what is expected of PE teachers to do during class periods may increase the awareness of the multi-faceted school community to the teachers’ current-relevant tasks. Of particular interest among the school stakeholders are the PE students who will be teaching the subject in elementary and high schools upon completion the PE preparation program. It is interesting to analyze how they perceive their own future core tasks compared with those perceptions of the other PE-relevant school stakeholders.

## 2 Methods

### 2.1 Participants

Three-hundred and eighty-one (381) participants [mean age = 35.5 years; standard deviation (SD) = 13.7] took part in the study (Table 1). These included 222 females (58.3%) and 157 males (41.2%). The participants were asked to select one or more of the following belonging/affiliation groups in order to determine their professional/occupational status: PE college students (future PE teachers), PE teachers, sport/physical activity coaches, sport/physical activity policymakers, and parents to children in the school system. The participants were informed that they could select more than one group. Each stakeholder group was composed of the number of participants who selected the given group. As can be seen in Table 1, the two most prevalent groups were PE students (205 participants; 53.8%) and PE teachers (114; 29.9%).

**Table 1:** The distribution of study participants according to stakeholder group, gender, and age [mean (SD)].

Group	Total		Males		Females	
	*n*	Age Mean (SD)	*n* (%)	Age Mean (SD)	*n* (%)	Age Mean (SD)
PE students	205	26.94 (7.41)	84 (41.0)	26.59 (6.53)	120 (58.5)	27.18 (7.98)
PE teachers	114	43.15 (11.81)	33 (28.9)	43.88 (11.36)	80 (70.2)	42.86 (12.05)
Coaches	102	36.59 (13.01)	57 (55.9)	37.11 (13.05)	45 (44.1)	35.93 (13.09)
Policymakers	107	34.21 (12.50)	41 (38.3)	35.77 (15.20)	66 (61.7)	33.27 (10.54)
Parents	100	46.65 (9.27)	32 (32.0)	47.94 (9.42)	66 (66.0)	46.02 (9.20)
Total	381*	35.52 (13.74)	157 (41.2)	35.37 (14.09)	222 (58.3)	35.63 (13.51)

PE, physical education; SD, standard deviation.

* 381 participants took part in the study; participants could select more than one group.

The number of group selections and their distribution made by the participants are presented in Table 2. About 54% of the participants selected only one group, while about 17% of them selected two groups and a similar percentage of participants selected three groups. Among those who selected only one group were 111 PE students (who were part of the total of 205 participants who selected this group), 31 PE teachers (part of 114), 14 coaches (part of 102), 12 policymakers (part of 107), and 38 parents (part of 100).

**Table 2:** The distribution of the groups selection made by the participants.

Number of selected groups	Number of selections	%	Groups’ %
0	20	5.2	5.2
1	206	54.1	59.3
2	66	17.3	76.6
3	67	17.6	94.2
4	21	5.5	99.7
5	1	0.3	100

### 2.2 Questionnaire

The questionnaire was composed of three parts. Part 1 included a consent form. Participants were thanked for their participation in the study and were told that they could withdraw from the questionnaire completion at any time. Part 2 included questions on the demographic and professional status of the participant. Part 3 included two closed questions, each consisting of a list of 20 items reflecting tasks of the PE teacher. The first closed question addressed core tasks of PE teachers in elementary schools, and the second question listed core tasks of PE teachers in high schools.

The PE teachers’ tasks were extracted by the principal author based on a comprehensive review of related literature [e.g., (23–26)], with a focus on what is expected of teachers to do in PE classes, as well as what is expected of them to do in elementary and high schools in Israel. After the preparation of a list of 22 potential tasks of the PE teacher, the list was sent for evaluation and contextual validation to five experts in the field of PE. Among the experts, two were researchers and senior lecturers who worked at a college which trains students to be PE teachers. Three of the experts were instructors in sport classes (e.g., track-and-field, volleyball) and sport pedagogy classes in a teacher's training program held by an academic college that focuses on PE training programs. Two of the instructors additionally served as supervisors of the students in their practical work at elementary and high schools. All experts had teaching experience of at least 20 years at a college level. In addition, all experts started their careers as PE teachers and thus had experience working in the educational system in Israel. The experts were asked to review the proposed list of the PE teacher's tasks and to add, and/or remove, and/or change any item. The comments made by the experts were reviewed by the principal author, who made the required revisions. The revised list of tasks was sent one more time to the experts who reviewed it and approved the revisions made.

The finalized list was composed of 20 tasks, as can be seen in the Appendix. Three types of items were included: health-oriented items (e.g., Items 1, 2, 14, 17), sport-oriented items (e.g., Items 3, 4, 6, 10), and school students’ self-development-oriented items (e.g., Items 5, 9, 13, 20).

### 2.3 Procedure

The study was approved by the Ethics Committee of the Levinsky-Wingate Academic College (approval number 438). The questionnaire was distributed to the participants via two main online vehicles:

(a) The online intranet network of the PE students at the Levinsky-Wingate Academic College. This network included all students who were enrolled in the program at the time of recruitment (circa 1,270 students, including circa 1,000 undergraduate and circa 200 graduate students). Additionally, the network included individuals studying towards their PE teaching certification (circa 70 students). Sixteen percent of the students (205) who were part of the online intranet network completed the questionnaire.
(b) Online social media platforms of PE teachers (e.g., Facebook, WhatsApp) who work at elementary and high schools in Israel. This platform was designed for PE teachers to share instructional tips, ideas for lesson plans, practical experiences, and pedagogical challenges they face in their daily teaching.

The participants were asked to select the five most important core tasks of the PE teacher in elementary schools and the five most important tasks of the PE teacher in high schools from the 20 listed tasks. The participants were not required to rank the selected tasks from the most important one to the less important one.

### 2.4 Statistical analysis

Chi-square tests of independence were conducted to examine the associations between the categorical variables, namely to study differences in the participants’ perceptions of the PE teacher's tasks. The Chi-square analyses were separately conducted for the tasks of PE teachers in elementary schools and high schools. In addition, Chi-square analyses were used to examine differences in the participants’ perceptions according to the number of groups’ selections they made, namely participants who selected one group, two groups, three groups, and four groups.

Effect sizes were calculated using Cramér's V—a standardized measure of association for nominal variables based on the chi-square statistic, providing an index of the strength of the relationships between the categories. Values of Cramér's V range from 0 (no association) to 1 (perfect association), with higher values indicating a stronger association. The effect size was computed for each chi-square test to allow for a more comprehensive interpretation of the findings, independent of sample size. The interpretation of Cramér's V values followed Cohen's (1988) (27) conventional benchmarks. Data were analyzed using SPSS version 29. Statistical significance was set at an *α* of 0.05 for all statistical tests.

## 3 Results

We first present the descriptive analyses of the perceptions of the participants as a total group. This is then followed by the analysis of the perceptions of each of the five groups of stakeholders separately.

### 3.1 The core tasks of the PE teacher according to the participants’ perceptions

The total group of participants’ perceptions of the core tasks of PE teachers are presented in Table 3: their perceptions of the PE teachers’ tasks in elementary schools (left panel), their perceptions of the PE teachers’ tasks in high schools (middle panel), and their perceptions of the PE teachers’ tasks for the combined elementary and high school teachers (right panel).

**Table 3:** The participants’ perceptions of tasks of PE teachers in elementary schools, high schools, and elementary and high schools combined (all). The table presents the ranks of the selections, the number and percentage of selections of each task, and values of statistical analyses.

PE Tasks	Elementary School			High School			Statistical values			All		
	Rank	Yes	No	Rank	Yes	No	*χ*²	*P* Value	ES	Rank	Yes	No
		*n* (%)	*n* (%)		*n* (%)	*n* (%)					*n* (%)	*n* (%)
Encouraging a healthy and active lifestyle	3	197 (51.7%)	184 (48.3%)	1	225 (59.1%)	156 (40.9%)	4.16*	0.04	0.07	1	442 (55.4%)	340 (44.6%)
Promoting engagement in physical activity	1	212 (55.6%)	169 (44.4%)	2	195 (51.2%)	186 (48.8%)	1.52	0.22	0.05	2	407 (53.4%)	355 (46.6%)
Teaching a range of sport disciplines	2	209 (54.9%)	172 (45.1%)	6	133 (34.9%)	248 (65.1%)	30.64**	<0.001	0.20	3	342 (44.9%)	420 (55.1%)
Developing sport skills	5	160 (42.0%)	221 (58.0%)	3	166 (43.6%)	215 (56.4%)	0.19	0.66	0.02	4	326 (42.8%)	436 (57.2%)
Enhancing children's self-confidence	4	188 (49.3%)	193 (50.7%)	5	134 (35.2%)	247 (64.8%)	15.68**	<0.001	0.14	5	322 (42.3%)	440 (57.7%)
Encouraging involvement in sport	8	101 (26.5%)	280 (73.5%)	4	145 (38.1%)	236 (61.9%)	11.62**	<0.001	0.12	6	246 (32.3%)	516 (67.7%)
Introducing a variety of physical activities	7	121 (31.8%)	260 (68.2%)	7	92 (24.1%)	289 (75.9%)	5.48*	0.02	0.09	7	213 (28.0%)	549 (72.0%)
Enhancing motor skills	6	125 (32.8%)	256 (67.2%)	12.5	73 (19.2%)	308 (80.8%)	18.45**	<0.001	0.16	8	198 (26.0%)	564 (74.0%)
Instilling social and cultural values	9	90 (23.6%)	291 (76.4%)	9	83 (21.8%)	298 (78.2%)	0.37	0.55	0.02	9	173 (22.7%)	589 (77.3%)
Shaping children according to sport-related values	11	76 (19.9%)	305 (80.1%)	8	87 (22.8%)	294 (77.2%)	0.94	0.33	0.04	10	163 (21.4%)	599 (78.6%)
Educating through physical empowerment	10	82 (21.5%)	299 (78.5%)	11	76 (19.9%)	305 (80.1%)	0.29	0.59	0.02	11	158 (20.7%)	604 (79.3%)
Supporting children's mental health	12	75 (19.7%)	306 (80.3%)	10	81 (21.3%)	300 (78.7%)	0.29	0.59	0.02	12	156 (20.5%)	606 (79.5%)
Fostering self-esteem	13	66 (17.3%)	315 (82.7%)	12.5	73 (19.2%)	308 (80.8%)	0.43	0.51	0.02	13	139 (18.2%)	623 (81.8%)
Promoting health and well-being	16	37 (9.7%)	344 (90.3%)	14	50 (13.1%)	331 (86.9%)	2.19	0.14	0.05	14	87 (11.4%)	675 (88.6%)
Supporting developmental processes and motor skill acquisition	14	62 (16.3%)	319 (83.7%)	18.5	23 (6.0%)	358 (94.0%)	20.14**	<0.001	0.16	15	85 (11.2%)	677 (88.8%)
Creating opportunities for self-expression and creativity	16	37 (9.7%)	344 (90.3%)	16	39 (10.2%)	342 (89.8%)	0.06	0.81	0.01	16	76 (10.0%)	686 (90.0%)
Improving children's body composition	18	24 (6.3%)	357 (93.7%)	15	47 (12.3%)	334 (87.7%)	8.22**	0.00	0.10	17	71 (9.3%)	691 (90.7%)
Delivering knowledge and deepening understanding	20	21 (5.5%)	360 (94.5%)	17	35 (9.2%)	346 (90.8%)	3.78*	0.05	0.07	18	56 (7.3%)	706 (92.7%)
Fostering the development of physical literacy	17	25 (6.6%)	356 (93.4%)	18.5	23 (6.0%)	358 (94.0%)	0.09	0.77	0.01	19	48 (6.3%)	714 (93.7%)
Addressing individual needs	19	23 (6.0%)	358 (94.0%)	20	22 (5.8%)	359 (94.2%)	0.02	0.88	0.01	20	45 (5.9%)	717 (94.1%)

* *p* ≤ .05.

** *p* ≤ .01.

As can be seen in Table 3, the most frequently selected tasks were encouraging a healthy and active lifestyle (selected by 55.4% of the participants), promoting engagement in PA (53.4%), teaching a range of sport disciplines (44.9%), developing sport skills (42.8%), and enhancing children's self-confidence (42.3%). Among the least-selected tasks were creating opportunities for self-expression and creativity (10%), improving children's body composition (9.3%), delivering knowledge and deepening understanding (7.3%), fostering the development of physical literacy (6.3%), and addressing individual needs (5.9%).

Four of the top five selected tasks in elementary schools are aligned with those selected for all schools. However, the task of introducing a variety of PAs, which was ranked second for elementary schools (54.9%) was not included in the top five tasks in the ranking for all schools. Instead, the task of teaching a range of sport disciplines, which was ranked third for all schools, was not among the top selected five tasks for elementary schools. The least selected tasks were similar for all schools and elementary schools.

In high schools, four of the five most commonly selected tasks were similar to those ranked at the top of the list in elementary schools. The task of encouraging involvement in sport, which was ranked fourth for high schools (38.1%), was not selected among the top five tasks for elementary schools. However, the task of introducing a variety of PAs, which was selected second for elementary schools, was not included in the top five tasks for high schools. The least selected tasks for high schools were the same as the ones least selected for elementary schools, with one exception: the task of supporting developmental processes and motor skill acquisition (6%) did not appear among the least selected tasks for elementary schools.

### 3.2 Participants’ perceptions according to the stakeholder groups

The Chi-square analyses of the participants’ perceptions of the top tasks of PE teachers in elementary and high schools are presented in Tables 4, 5 by group—PE students, PE teachers, coaches, policymakers, and parents. The most selected tasks that were further analyzed were encouraging a healthy and active lifestyle, promoting engagement in PA, teaching a range of sport disciplines, developing sport skills, enhancing children's self-confidence, and encouraging involvement in sport.

**Table 4:** The participants’ perceptions by stakeholder groups—PE students, PE teachers, coaches, policymakers, and parents.

Groups	Elementary School		High School		Statistical values			Elementary School		High School		Statistical values		
	Yes (*n*, %)	No (*n*, %)	Yes (*n*, %)	No (*n* ,%)	*χ* ^2^	P	ES	Yes (*n*, %)	No (*n*, %)	Yes (*n*, %)	No (*n*, %)	χ^2^	P	ES
PE Students	Yes = 205							No = 176						
Encouraging a healthy and active lifestyle	102 (49.8%)	103 (50.2%)	109 (53.2%)	96 (46.8%)	0.48	0.49	0.03	95 (54.0%)	81 (46.0%)	116 (65.9%)	60 (34.1%)	5.22*	0.02	0.12
Promoting engagement in physical activity	120 (58.5%)	85 (41.5%)	112 (54.6%)	93 (45.4%)	0.64	0.43	0.04	92 (52.3%)	84 (47.7%)	83 (47.2%)	93 (52.8%)	0.92	0.34	0.05
Teaching a range of sport disciplines	117 (57.1%)	88 (42.9%)	61 (29.8%)	144 (70.2%)	31.14**	0.00	0.28	92 (52.3%)	84 (47.7%)	72 (40.9%)	104 (59.1%)	4.57*	0.03	0.11
Developing sport skills	85 (41.5%)	120 (58.5%)	99 (48.3%)	106 (51.7%)	1.93	0.16	0.07	75 (42.6%)	101 (57.4%)	67 (38.1%)	109 (61.9%)	0.76	0.38	0.05
Enhancing children's self-confidence	97 (47.3%)	108 (52.7%)	76 (37.1%)	129 (62.9%)	4.41*	0.04	0.10	91 (51.7%)	85 (48.3%)	58 (33.0%)	118 (67.0%)	12.67**	0.00	0.19
Encouraging involvement in sport	58 (28.3%)	147 (71.7%)	77 (37.6%)	128 (62.4%)	3.987*	0.05	0.10	43 (24.4%)	133 (75.6%)	68 (38.6%)	108 (61.4%)	8.22	0.00	0.15
PE Teachers	Yes = 114							No = 267						
Encouraging a healthy and active lifestyle	65 (57.0%)	49 (43.0%)	75 (65.8%)	39 (34.2%)	1.85	0.17	0.09	132 (49.4%)	135 (50.6%)	150 (56.2%)	117 (43.8%)	2.44	0.12	0.07
Promoting engagement in physical activity	56 (49.1%)	58 (50.9%)	61 (53.5%)	53 (46.5%)	0.44	0.51	0.04	156 (58.4%)	111 (41.6%)	134 (50.2%)	133 (49.8%)	3.65	0.06	0.08
Teaching a range of sport disciplines	73 (64.0%)	41 (36.0%)	50 (43.9%)	64 (56.1%)	9.34	0.00	0.20	136 (50.9%)	131 (49.1%)	83 (31.1%)	184 (68.9%)	21.74*High School	0.00	0.20
Developing sport skills	46 (40.4%)	68 (59.6%)	50 (43.9%)	64 (56.1%)	0.29	0.59	0.04	114 (42.7%)	153 (57.3%)	116 (43.4%)	151 (56.6%)	0.03	0.86	0.01
Enhancing children's self-confidence	57 (50.0%)	57 (50.0%)	41 (36.0%)	73 (64.0%)	4.58*	0.03	0.14	131 (49.1%)	136 (50.9%)	93 (34.8%)	174 (65.2%)	11.1**	0.00	0.14
Encouraging involvement in sport	23 (20.2%)	91 (79.8%)	40 (35.1%)	74 (64.9%)	6.34**	0.01	0.17	78 (29.2%)	189 (70.8%)	105 (39.3%)	162 (60.7%)	6.06	0.01	0.11
Coaches	Yes = 102							No = 279						
Encouraging a healthy and active lifestyle	52 (51.0%)	50 (49.0%)	54 (52.9%)	48 (47.1%)	0.08	0.78	0.02	145 (52.0%)	134 (48.0%)	171 (61.3%)	108 (38.7%)	4.93*	0.03	0.09
Promoting engagement in physical activity	50 (49.0%)	52 (51.0%)	47 (46.1%)	55 (53.9%)	0.18	0.67	0.03	162 (58.1%)	117 (41.9%)	148 (53.0%)	131 (47.0%)	1.42	0.23	0.05
Teaching a range of sport disciplines	60 (58.8%)	42 (41.2%)	37 (36.3%)	66 (64.7%)	10.78**	0.00	0.23	149 (53.4%)	130 (46.6%)	97 (34.8%)	182 (65.2%)	19.66**	0.00	0.19
Developing sport skills	32 (31.4%)	70 (68.6%)	43 (42.2%)	59 (57.8%)	2.55	0.11	0.11	128 (45.9%)	151 (54.1%)	123 (44.1%)	156 (55.9%)	0.18	0.67	0.02
Enhancing children's self-confidence	52 (51.0%)	50 (49.0%)	35 (34.3%)	67 (65.7%)	5.79*	0.02	0.17	136 (48.7%)	143 (51.3%)	99 (35.5%)	180 (64.5%)	10.06**	0.00	0.13
Encouraging involvement in sport	38 (37.3%)	64 (62.7%)	38 (37.3%)	64 (62.7%)	0.00	1.00	0.00	63 (22.6%)	216 (77.4%)	107 (38.4%)	172 (61.6%)	16.38	0.00	0.17
Policymakers	Yes = 107							No = 274						
Encouraging a healthy and active lifestyle	59 (55.1%)	48 (44.9%)	58 (54.2%)	49 (45.8%)	0.02	0.89	0.01	138 (50.4%)	136 (49.6%)	167 (60.9%)	107 (39.1%)	6.22**	0.01	0.11
Promoting engagement in physical activity	58 (54.2%)	49 (45.8%)	50 (46.7%)	57 (53.3%)	1.20	0.27	0.08	154 (56.2%)	120 (43.8%)	145 (52.9%)	129 (47.1%)	0.60	0.44	0.03
Teaching a range of sport disciplines	63 (58.9%)	44 (41.1%)	36 (33.6%)	66 (61.7%)	11.65**	0.00	0.24	146 (53.3%)	128 (46.7%)	96 (35.0%)	178 (65.0%)	18.50**	0.00	0.18
Developing sport skills	37 (34.6%)	70 (65.4%)	53 (49.5%)	54 (50.5%)	4.91*	0.03	0.15	123 (44.9%)	151 (55.1%)	113 (41.2%)	161 (58.8%)	0.74	0.39	0.04
Enhancing children's self-confidence	50 (46.7%)	57 (53.3%)	34 (31.8%)	73 (68.2%)	5.02*	0.03	0.15	138 (50.4%)	136 (49.6%)	100 (36.5%)	174 (63.5%)	10.73	0.00	0.14
Encouraging involvement in sport	26 (24.3%)	81 (75.7%)	34 (31.8%)	73 (68.2%)	1.48	0.22	0.08	75 (27.4%)	199 (72.6%)	111 (40.5%)	163 (59.5%)	10.55	0.00	0.14
Parents	Yes = 100							No = 281						
Encouraging a healthy and active lifestyle	52 (52.0%)	48 (48.0%)	62 (62.0%)	38 (38.0%)	2.04	0.15	0.10	145 (51.6%)	136 (48.4%)	163 (58.0%)	118 (42.0%)	2.33	0.13	0.06
Promoting engagement in physical activity	55 (55.0%)	45 (45.0%)	48 (48.0%)	52 (52.0%)	0.98	0.32	0.07	157 (55.9%)	124 (44.1%)	147 (52.3%)	134 (47.7%)	0.72	0.40	0.04
Teaching a range of sport disciplines	49 (49.0%)	51 (51.0%)	37 (37.0%)	63 (63.0%)	2.94	0.09	0.12	160 (56.9%)	121 (43.1%)	96 (34.2%)	185 (65.8%)	29.39**	0.00	0.23
Developing sport skills	43 (43.0%)	57 (57.0%)	40 (40.0%)	60 (60.0%)	0.19	0.67	0.03	117 (41.6%)	164 (58.4%)	126 (44.8%)	155 (55.2%)	0.59	0.44	0.03
Enhancing children's self-confidence	55 (55.0%)	45 (45.0%)	30 (30.0%)	70 (70.0%)	12.79**	0.00	0.25	133 (47.3%)	148 (52.7%)	104 (37.0%)	177 (63.0%)	6.14**	0.01	0.10
Encouraging involvement in sport	24 (24.0%)	76 (76.0%)	33 (33.0%)	67 (67.0%)	1.99	0.16	0.10	77 (27.4%)	204 (72.6%)	112 (39.9%)	169 (60.1%)	9.77**	0.00	0.13

* *p* ≤ 0.05.

** *p* ≤ 0.01.

**Table 5:** The participants’ perceptions by stakeholder groups—PE students, PE teachers, coaches, policymakers, and parents—a comparison between the most selected tasks of the participants in a given group and the most selected tasks of those who did not belong to this given group. .

Group	Elementary School			High School		
PE Students: Yes = 205, No = 176	χ^2^	P	ES	χ^2^	P	ES
Encouraging a healthy and active lifestyle	0.68	0.41	0.04	6.36**	0.01	0.13
Promoting engagement in physical activity	1.51	0.22	0.06	2.12	0.15	0.08
Teaching a range of sport disciplines	0.88	0.35	0.05	2.93	0.09	0.09
Developing sport skills	0.05	0.82	0.01	9.40**	0.00	0.15
Enhancing children's self-confidence	0.73	0.39	0.04	1.79	0.18	0.07
Encouraging involvement in sport	0.73	0.39	0.04	4.29**	0.04	0.10
PE Teachers: Yes = 114, No = 267	χ^2^	P	ES	χ^2^	P	ES
Encouraging a healthy and active lifestyle	1.84	0.18	0.07	1.94	0.16	0.07
Promoting engagement in physical activity	2.80	0.09	0.09	0.28	0.60	0.03
Teaching a range of sport disciplines	5.54**	0.02	0.12	5.50*	0.02	0.12
Developing sport skills	0.18	0.67	0.02	0.05	0.83	0.01
Enhancing children's self-confidence	0.03	0.87	0.01	0.13	0.72	0.02
Encouraging involvement in sport	3.35	0.07	0.09	0.28	0.60	0.03
Coaches: Yes = 102, No = 279	χ^2^	P	ES	χ^2^	P	ES
Encouraging a healthy and active lifestyle	0.03	0.86	0.01	2.27	0.13	0.08
Promoting engagement in physical activity	2.48	0.12	0.08	1.61	0.20	0.07
Teaching a range of sport disciplines	0.89	0.35	0.05	0.01	0.91	0.01
Developing sport skills	6.45**	0.01	0.13	0.04	0.84	0.01
Enhancing children's self-confidence	0.15	0.70	0.02	0.12	0.73	0.02
Encouraging involvement in sport	8.26**	0.00	0.15	0.18	0.68	0.02
Policymakers: Yes = 107, No = 274	χ^2^	P	ES	χ^2^	P	ES
Encouraging a healthy and active lifestyle	0.70	0.40	0.04	0.61	0.43	0.04
Promoting engagement in physical activity	0.13	0.72	0.02	0.85	0.36	0.05
Teaching a range of sport disciplines	0.97	0.32	0.05	0.04	0.84	0.01
Developing sport skills	3.36	0.07	0.09	1.68	0.19	0.07
Enhancing children's self-confidence	0.41	0.52	0.03	0.64	0.43	0.04
Encouraging involvement in sport	0.37	0.54	0.03	2.05	0.15	0.07
Parents: Yes = 100, No = 281	χ^2^	P	ES	χ^2^	P	ES
Encouraging a healthy and active lifestyle	0.01	0.95	0.004	0.49	0.49	0.04
Promoting engagement in physical activity	0.02	0.88	0.01	0.55	0.46	0.04
Teaching a range of sport disciplines	1.88	0.17	0.07	0.11	0.74	0.02
Developing sport skills	0.06	0.81	0.012	0.70	0.40	0.04
Enhancing children's self-confidence	1.74	0.19	0.07	1.59	0.21	0.07
Encouraging involvement in sport	0.44	0.51	0.03	1.47	0.23	0.06

* *p* ≤ 0.05.

** *p* ≤ 0.01.

The main purpose of these analyses was to examine whether participants in a given stakeholder group and those who were not in this group differed in their selection of tasks for teachers in elementary vs. high schools. More specifically, four analyses were performed. One, a comparison between the most selected tasks of the participants within a given stakeholder group. Two, a comparison between the most selected tasks of the participants who did not select this given group. Three, a comparison between the most selected tasks of the participants in a given group and those who did not belong to this given group for PE teachers in elementary schools. The analyses of these three comparisons are presented in Table 4. Four, a comparison between the most selected tasks of the participants in a given group and those who did not belong to this given group (see Table 5). Analyses are discussed below for each group of stakeholders separately.

#### 3.2.1 PE students/no-PE students

The students expected PE teachers in elementary schools to put more emphasis on teaching a range of sport disciplines and enhancing children's self-confidence. In addition, they thought that PE teachers in high schools should encourage involvement in sport. Those who were not students would like to see the elementary school's PE teacher teach a range of sport disciplines and enhance children's self-confidence. For the PE teachers in high schools, they preferred that teachers encourage a healthy and active lifestyle and encourage involvement in sport.

In a comparison between students and no-students, three tasks were perceived differently, all were associated with core tasks of teachers in high schools. The first task to be different between the groups was encouraging a healthy and active lifestyle, which was higher in the no-students group. The second task was teaching a range of sport disciplines, which was also higher in the no-students group. The third task was developing sport skills, which was higher in the student group.

#### 3.2.2 PE teachers/no-PE teachers

The teachers expected PE teachers in elementary schools to put an emphasis on teaching a range of sport disciplines and on enhancing children's self-confidence. However, they preferred that PE teachers in high schools encourage involvement in sport. The no-teachers group expected PE teachers in elementary schools to teach a range of sport disciplines and enhance children's self-confidence. These two tasks are the same as those stressed by the students and teacher's stakeholder's groups. For the PE teachers in high schools, the no-teachers group outlined encouraging involvement in sport as an important task. In a comparison between teachers and no-teachers, both viewed the task of teaching a range of sport disciplines as particularly essential in both elementary and high schools.

#### 3.2.3 Coaches/no-coaches

The coaches expected PE teachers in elementary schools to focus on two tasks: teaching a range of sport disciplines and enhancing children's self-confidence. Similarly, the no-coaches group expected PE teachers in elementary schools to teach a range of sport disciplines and enhance children's self-confidence. These two tasks are the same as the ones selected by the students and teacher's stakeholder groups. For the PE teachers in high schools, the no-coaches group preferred two tasks: encouraging a healthy and active lifestyle and encouraging involvement in sport.

In a comparison between coaches and no-coaches, two tasks were found to be different, both in elementary school teachers. Coaches rated higher the task of encouraging involvement in sport, while those who were not coaches rated higher the role of developing sport skills.

#### 3.2.4 Policymakers/no-policymakers

The policymakers expected PE teachers in elementary schools to put an emphasis on teaching a range of sport disciplines and on enhancing children's self-confidence. Similarly, the no-policymakers group would like to see PE teachers in elementary schools teach a range of sport disciplines and enhance children's self-confidence. These two tasks are the same as the ones selected by the students, teachers, and coaches’ stakeholder's groups. For PE teachers in high schools, the group of the no-policymakers stressed two tasks: encouraging a healthy and active lifestyle and encouraging involvement in sport. In a comparison between those who were policymakers and those who were not policymakers, no task was found to be different.

#### 3.2.5 Parents/no-parents

The parents expected PE teachers in elementary schools to focus on enhancing children's self-confidence. The no-parents group expected the PE teachers in elementary schools to teach a range of sport disciplines and enhance children's self-confidence. These two tasks were the same as the tasks ranked by the student, teacher and policymaker groups. For the PE teachers in high schools, the no-parents group emphasized one task: encouraging involvement in sport. In a comparison between those who were parents and those who were not parents, no task was found to be different.

### 3.3 Participants’ perceptions according to the number of stakeholder group selections

The analyses of the participants’ perceptions according to the number of the stakeholder group selections they made are presented in Table 6 (the core tasks of the PE teacher in elementary schools), 7 (the core tasks of the PE teacher in high schools), and 8 (the core tasks of the PE teacher in elementary schools and high schools combined). The most prominent finding that emerged from these analyses was that no significant differences were found in the participants’ perceptions of the top selected core tasks of the PE teacher. In other words, those who selected one (stakeholder) group, two groups, three groups, or four groups perceived the core tasks of the PE teachers in a similar manner. As can be seen in Tables 6–8, several differences in the participants’ perceptions did exist; however, none of them was among the top selected tasks.

**Table 6:** The participants’ perceptions of the core tasks of the PE teacher in elementary schools according to the number of the stakeholder group selections they made.

Number of selections of the stakeholder groups	1 *n* = 206		2 *n* = 66		3 *n* = 67		4 *n* = 21		Statistical values		
Elementary school	Rank	Yes(%)	Rank	Yes(%)	Rank	Yes(%)	Rank	Yes(%)	χ^2^	P	ES
Developing sport skills	5	98 (47.6%)	6.5	23 (34.8%)	6	25 (37.3%)	10.5	6 (28.6%)	6.15	0.10	0.13
Enhancing motor skills	7	63 (30.6%)	8.5	22 (33.3%)	5	28 (41.8%)	7	7 (33.3%)	2.86	0.41	0.09
Learning different sport disciplines	6	69 (33.5%)	5	26 (39.4%)	10.5	15 (22.4%)	12	4 (19.0%)	6.35	0.10	0.13
Teaching a range of sport disciplines	2	109 (52.9%)	3	34 (51.5%)	1	44 (65.7%)	1.5	12 (57.1%)	3.80	0.28	0.10
Promoting engagement in physical activity	1	121 (58.7%)	1	37 (56.1%)	2	37 (55.2%)	7	7 (33.3%)	5.02	0.17	0.12
Encouraging a healthy and active lifestyle	3	102 (49.5%)	2	35 (53.0%)	4	35 (52.2%)	1.5	12 (57.1%)	0.65	0.89	0.04
Educating through physical empowerment	11	36 (17.5%)	10	20 (30.3%)	10.5	15 (22.4%)	7	7 (33.3%)	6.74	0.08	0.14
Enhancing children's self-confidence	4	100 (48.5%)	4	31 (47.0%)	3	36 (53.7%)	3	9 (42.9%)	1.04	0.79	0.05
Improving children's body composition	17	16 (7.8%)	20	2 (3.0%)	18.5	4 (6.0%)	19	0 (0.0%)	3.45	0.33	0.10
Shaping children according to sport-related values	10	42 (20.4%)	13	11 (16.7%)	14	10 (14.9%)	10.5	6 (28.6%)	2.44	0.49	0.08
Supporting children's mental health	12	35 (17.0%)	12	14 (21.2%)	7	19 (28.4%)	15.5	2 (9.5%)	5.64	0.13	0.13
Supporting developmental processes and motor skill acquisition	13.5	25 (12.1%)	15	8 (12.1%)	9	17 (25.4%)	4	8 (38.1%)	14.95**	0.00	0.20
Promoting health and well-being	16	23 (11.2%)	17	4 (6.1%)	18.5	4 (6.0%)	13	3 (14.3%)	3.12	0.37	0.09
Encouraging involvement in sport	8	51 (24.8%)	6.5	23 (34.8%)	12	13 (19.4%)	7	7 (33.3%)	4.94	0.18	0.12
Fostering the development of physical literacy	19	9 (4.4%)	17	4 (6.1%)	15.5	8 (11.9%)	15.5	2 (9.5%)	5.22	0.16	0.12
Fostering self-esteem	15	24 (11.7%)	8.5	22 (33.3%)	13	12 (17.9%)	15.5	2 (9.5%)	17.78**	0.00	0.22
Creating opportunities for self-expression and creativity	13.5	25 (12.1%)	19	3 (4.5%)	15.5	8 (11.9%)	19	0 (0.0%)	5.84	0.12	0.13
Instilling social and cultural values	9	48 (23.3%)	11	15 (22.7%)	8	18 (26.9%)	7	7 (33.3%)	1.36	0.71	0.06
Addressing individual needs	19	9 (4.4%)	14	10 (15.2%)	20	3 (4.5%)	19	0 (0.0%)	12.17**	0.01	0.18
Delivering knowledge and deepening understanding	19	9 (4.4%)	17	4 (6.1%)	17	6 (9.0%)	15.5	2 (9.5%)	2.52	0.47	0.08

1—one group selection.

2—two groups selection.

3—three groups selection.

4—four groups selection.

** *p* < 0.01.

**Table 7:** The participants’ perceptions of the core tasks of the PE teacher in high schools according to the number of the stakeholder group selections they made.

Number of selections of the stakeholder groups	1 *n* = 206		2 *n* = 66		3 *n* = 67		4 *n* = 21		Statistical values		
High school	Rank	Yes(%)	Rank	Yes(%)	Rank	Yes(%)	Rank	Yes(%)	χ^2^	*P*	ES
Developing sport skills	3	84 (40.8%)	2	29 (43.9%)	3	34 (50.7%)	3	9 (42.9%)	2.06	0.56	0.08
Enhancing motor skills	11	36 (17.5%)	13	11 (16.7%)	11	15 (22.4%)	8.5	5 (23.8%)	1.35	0.72	0.06
Learning different sport disciplines	7	54 (26.2%)	11	14 (21.2%)	10	16 (23.9%)	10.5	4 (19.0%)	1.07	0.79	0.05
Teaching a range of sport disciplines	6	70 (34.0%)	4.5	23 (34.8%)	4.5	24 (35.8%)	5	7 (33.3%)	0.09	0.99	0.02
Promoting engagement in physical activity	2	112 (54.4%)	3	28 (42.4%)	2	35 (52.2%)	2	10 (47.6%)	2.99	0.39	0.09
Encouraging a healthy and active lifestyle	1	127 (61.7%)	1	37 (56.1%)	1	36 (53.7%)	1	11 (52.4%)	1.96	0.58	0.07
Educating through physical empowerment	13	34 (16.5%)	8.5	16 (24.2%)	7	20 (29.9%)	16	2 (9.5%)	7.82*	0.05	0.15
Enhancing children's self-confidence	5	78 (37.9%)	7	19 (28.8%)	4.5	24 (35.8%)	5	7 (33.3%)	1.85	0.61	0.07
Improving children's body composition	15	28 (13.6%)	15	8 (12.1%)	17	6 (9.0%)	13	3 (14.3%)	1.06	0.79	0.05
Shaping children according to sport-related values	8	48 (23.3%)	8.5	16 (24.2%)	13	13 (19.4%)	13	3 (14.3%)	1.37	0.71	0.06
Supporting children's mental health	9	42 (20.4%)	11	14 (21.2%)	9	18 (26.9%)	16	2 (9.5%)	3.09	0.38	0.09
Supporting developmental processes and motor skill acquisition	18	13 (6.3%)	19	5 (7.6%)	19.5	2 (3.0%)	19	1 (4.8%)	1.48	0.69	0.06
Promoting health and well-being	14	31 (15.0%)	15	8 (12.1%)	18	5 (7.5%)	10.5	4 (19.0%)	3.20	0.36	0.09
Encouraging involvement in sport	4	83 (40.3%)	4.5	23 (34.8%)	7	20 (29.9%)	5	7 (33.3%)	2.68	0.44	0.09
Fostering the development of physical literacy	20	11 (5.3%)	20	2 (3.0%)	14.5	8 (11.9%)	19	1 (4.8%)	5.34	0.15	0.12
Fostering self-esteem	10	40 (19.4%)	12	13 (19.7%)	12	14 (20.9%)	13	3 (14.3%)	0.45	0.93	0.04
Creating opportunities for self-expression and creativity	16	23 (11.2%)	17	7 (10.6%)	16	7 (10.4%)	16	2 (9.5%)	0.08	0.99	0.01
Instilling social and cultural values	12	35 (17.0%)	6	22 (33.3%)	7	20 (29.9%)	8.5	5 (23.8%)	10.02*	0.02	0.17
Addressing individual needs	19	12 (5.8%)	17	7 (10.6%)	19.5	2 (3.0%)	19	1 (4.8%)	3.56	0.31	0.10
Delivering knowledge and deepening understanding	17	15 (7.3%)	18	6 (9.1%)	14.5	8 (11.9%)	7	6 (28.6%)	10.30*	0.02	0.17

1—one group selection.

2—two groups selection.

3—three groups selection.

4—four groups selection.

* *p* < 0.05.

**Table 8:** The participants’ perceptions of the core tasks of the PE teacher in elementary schools and high schools combined according to the number of the stakeholder group selections they made.

Number of selections of the stakeholder groups	1 *n* = 206		2 *n* = 66		3 *n* = 67		4 *n* = 21		Statistical values		
ALL	Rank	yes (%)	Rank	yes (%)	Rank	yes (%)	Rank	yes (%)	χ^2^	Sig	ES
Developing sport skills	3	182 (44.2%)	4	52 (39.4%)	5	59 (44.0%)	5	15 (35.7%)	1.89	0.60	0.05
Enhancing motor skills	8	99 (24.0%)	11	33 (25.0%)	6	43 (32.1%)	7.5	12 (28.6%)	3.63	0.30	0.07
Learning different sport disciplines	7	123 (29.9%)	7	40 (30.3%)	11	31 (23.1%)	12.5	8 (19.0%)	4.29	0.23	0.08
Teaching a range of sport disciplines	4	179 (43.4%)	3	57 (43.2%)	3	68 (50.7%)	2	19 (45.2%)	2.36	0.50	0.06
Promoting engagement in physical activity	1	233 (56.6%)	2	65 (49.2%)	1	72 (53.7%)	3	17 (40.5%)	5.36	0.15	0.09
Encouraging a healthy and active lifestyle	2	229 (55.6%)	1	72 (54.5%)	2	71 (53.0%)	1	23 (54.8%)	0.28	0.96	0.02
Educating through physical empowerment	12	70 (17.0%)	9	36 (27.3%)	9	35 (26.1%)	10	9 (21.4%)	9.29*	0.03	0.11
Enhancing children's self-confidence	5	178 (43.2%)	5	50 (37.9%)	4	60 (44.8%)	4	16 (38.1%)	1.84	0.61	0.05
Improving children's body composition	16	44 (10.7%)	18	10 (7.6%)	18	10 (7.5%)	17.5	3 (7.1%)	2.16	0.54	0.05
Shaping children according to sport-related values	9	90 (21.8%)	13	27 (20.5%)	13	23 (17.2%)	10	9 (21.4%)	1.37	0.71	0.04
Supporting children's mental health	11	77 (18.7%)	12	28 (21.2%)	8	37 (27.6%)	16	4 (9.5%)	8.18*	0.04	0.11
Supporting developmental processes and motor skill acquisition	17	38 (9.2%)	15	13 (9.8%)	14	19 (14.2%)	10	9 (21.4%)	7.57	0.06	0.10
Promoting health and well-being	14	54 (13.1%)	16	12 (9.1%)	19	9 (6.7%)	14	7 (16.7%)	5.95	0.11	0.09
Encouraging involvement in sport	6	134 (32.5%)	6	46 (34.8%)	10	33 (24.6%)	6	14 (33.3%)	3.88	0.27	0.07
Fostering the development of physical literacy	20	20 (4.9%)	20	6 (4.5%)	15	16 (11.9%)	17.5	3 (7.1%)	9.49*	0.02	0.11
Fostering self-esteem	13	64 (15.5%)	10	35 (26.5%)	12	26 (19.4%)	15	5 (11.9%)	9.39*	0.02	0.11
Creating opportunities for self-expression and creativity	15	48 (11.7%)	18	10 (7.6%)	16	15 (11.2%)	19	2 (4.8%)	3.34	0.34	0.07
Instilling social and cultural values	10	83 (20.1%)	8	37 (28.0%)	7	38 (28.4%)	7.5	12 (28.6%)	6.42	0.09	0.09
Addressing individual needs	19	21 (5.1%)	14	17 (12.9%)	20	5 (3.7%)	20	1 (2.4%)	13.62**	0.00	0.14
Delivering knowledge and deepening understanding	18	24 (5.8%)	18	10 (7.6%)	17	14 (10.4%)	12.5	8 (19.0%)	10.97**	0.01	0.12

1—one group selection.

2—two groups selection.

3—three groups selection.

4—four groups selection.

* *p* ≤ 0.05

** *p* ≤ 0.01

## 4 Discussion

The findings of the current study highlighted the multifaceted task of the PE teacher at elementary and high school settings, as perceived by the five groups of the school stakeholders. Two main findings emerged: First, using various Chi-square tests we found substantial agreement among the different school stakeholder groups regarding the core tasks of PE teachers in elementary and high schools. The most selected six core tasks included: (a) encouraging a healthy and active lifestyle, (b) promoting engagement in PA, (c) teaching a variety of sport disciplines, (d) enhancing children's self-confidence, and (e) developing sport-specific skills. The second finding was that there were differences between the perceived tasks of PE teachers for children in elementary schools vs. for adolescents in high schools. Perceptions of these tasks also differed between the groups of the school stakeholders.

The core task most frequently selected by all the school stakeholder groups except for the student group, was encouraging a healthy and active lifestyle for adolescents (high school). This finding underscores the strong emphasis placed on this task by teachers, coaches, policymakers, and parents during the adolescent period. The selection of this task was also common for elementary school, although somewhat less than for high school.

While the present discussion emphasizes PA and sport participation as central components of a healthy and active lifestyle, it is important to acknowledge that health-related behaviors extend beyond these two domains. A healthy lifestyle also encompasses other factors, among them adequate sleep, balanced nutrition, stress management, and overall well-being. However, these aspects of a healthy and active lifestyle were not emphasized by the different school stakeholders who participated in the current study, and therefore did not emerge as central elements of the perceived core roles of the PE teacher. Consequently, the focus on PA and PE reflects the perspectives expressed by the school stakeholders and the specific context and objectives of the current research, while recognizing that health promotion in educational settings is inherently multifaceted.

Longitudinal evidence shows that PA tracks from both childhood and adolescence into adulthood. Results of tracking studies support the idea that enhancing PA during these early life stages is important for long-term public health promotion. Tracking appears to strengthen with age: stability is typically weaker in early childhood and becomes stronger in adolescence, indicating that adolescent activity patterns are more predictive of adult behavior (28).

A recent systematic review and meta-analysis further demonstrated that PA and sport participation track from both childhood and adolescence into adulthood, although correlations were generally small, indicating that early activity increased the likelihood of later activity without fully determining it (29). Another systemic review showed a positive association between motor competence and cardiorespiratory fitness and musculoskeletal fitness (30). The findings emerged from Cattuzzo et al.'s review confirmed that the trajectory and direction of one's activity behavior changes as they transition from adolescence into young adulthood (31).

Developing robust motor skills during childhood and adolescence plays a pivotal role in establishing a foundation for a healthy and active lifestyle. Fundamental movement skills (FMS), such as balancing, catching, jumping, running, and throwing, are considered “building blocks” for more complex sport- and activity-specific movements, enabling participation in a wide variety of PAs throughout life. A literature review by Lubans and colleagues (32) indicated strong evidence for a positive association between FMS competency and PA in children and adolescents. Children engaging in structured PA, either exclusively or in combination with unstructured PA was beneficial for children's gross motor development, whereas engaging in unstructured PA lacks such effectiveness. PA seems to be beneficial for motor skill development, particularly when implemented in a formal setting with guided opportunities for practice (24).

The finding that PE teachers are expected to encourage a healthy and active lifestyle aligns closely with public health goals and educational curricula worldwide [e.g., (6, 18, 33)]. According to the World Health Organization (WHO) (33), children and adolescents aged 5–17 years should engage in an average of at least 60 min per day of moderate to vigorous intensity aerobic PA throughout the week. In addition, vigorous-intensity activities, as well as those that strengthen muscles and bones, should be performed at least three times per week. However, according to the 2024 United States Report Card on Physical Activity for Children and Youth (34), only 20% to 28% of children and adolescents aged 6–17 years meet the recommended guideline of 60 min of daily PA. This proportion has not improved over the past decade.

The low compliance with PA guidelines needs to be addressed by professionals and policymakers in the fields of health, sport participation, and PE. The Institute of Medicine (34) highlights the essential task of schools and specifically PE programs in ensuring that children and adolescents engage in regular PA. Similarly to the WHO, the report of the Institute of Medicine recommends that all students participate in at least 60 min of moderate to vigorous PA daily, with PE serving as a key delivery mechanism. Moreover, the report emphasizes that certified PE teachers are critical to the success of this effort. PE teachers are not only instructors but also pivotal health and education advocates within the school system (34). The promotion of solid motor competence in childhood and adolescence serves as a long-term investment in public health (35).

Enhancing self-confidence emerged in our study as a highly selected core task of the PE teacher. This finding is in line with an expanded conceptualization of PE's contribution to the child's development, which extends beyond physical fitness and sport instruction to encompass emotional, psychological, and social development. Several review articles and studies provide support for this finding. For example, a review article by Bailey and colleagues (1) found that PE, as well as sport activities offered by the school, can foster self-concept, self-esteem, self-confidence, mood, and psychological well-being. Similarly, evidence from a systematic review by Eime and colleagues (36) underscored that participation in sport, particularly team-based activities, benefits children's psychological and social health by promoting self-esteem and social interaction. In another review article the authors stressed that “the deliberate preparation approach proposes that structured physical skill development during the early years could provide a situated learning environment for students to acquire the behavioral, psychological, and movement skills that increase the likelihood of lifelong physical activity” (10).

In addition, strong correlations were found among physical exercise, social-emotional competence, and self-esteem, suggesting that these jointly enhance social adaptability (37). In another study (38) it was found that incorporating social-emotional learning interventions into PE classes significantly improved personal and interpersonal development, including teamwork, self-awareness, and reflective abilities. The emphasis placed in the current study on psychological outcomes—in our case self-confidence—particularly by the parents, indicate that PE teachers are not only expected to promote PA, but also to nurture students’ well-being.

Interestingly, although various emotional and social developmental tasks, including instilling social and cultural values, supporting children's mental health, and fostering self-esteem, were recognized in our study, they were selected less frequently by the different groups of the school stakeholders. This finding may suggest that while these tasks are acknowledged, their benefit is often viewed as a secondary or indirect outcome of PE. However, evidence suggests that such dimensions should be considered central to PE (12, 13). In fact, neglecting these aspects may contribute to disengagement in PA among youth, especially those who struggle with traditional measures of athleticism.

From a practical point of view, we propose that the voice of the stakeholders be heard among policymakers and teachers in their attempts to develop a relevant PE evidence-based policy, reflecting the core tasks perceptions of the PE teacher. The policy should also account for current (e.g., promoting health and encouraging students to be physically active) coherent, inclusive, and pedagogically sound approaches of PE. Indeed, current health statements endorsed by the Ministry of Educations in Israel (21) already consider the perceptions of the stakeholders discussed in our study; however, dramatic changes in the PE policy have not yet been made.

It is important to emphasis that about 50% of the participants in our study selected health-oriented tasks as the main core tasks of the PE teachers. In other words, school stakeholders perceived the PE teacher as a health promotor. These perceptions are in line with health organizations’ [e.g., (22, 33)] expectations of PE teachers: encourage school students to develop an active and healthy lifestyle. However, PE policymakers and PE teachers should critically consider whether reinforcing a biomedical discourse truly enhances PE's educational legitimacy. In our study, among the six most selected tasks by the stakeholders were teaching a range of sport disciplines and developing sport skills. The pedagogical conflict between the importance of the health-oriented tasks and the sport-oriented tasks of the PE teachers still exists at the current time [see (2, 4, 5)].

Three limitations of our study should be outlined: First, the questionnaire given to the five groups of school stakeholders was reviewed by experts, but lacks psychometric validation. Although the list of tasks presented to the participants reflected the core tasks discussed in the PE literature, future versions of the questionnaire used in our study should exhibit psychometric values, such as reliability, internal validity, or construct validity.

Second, more than half of the participants (205) were PE students, ie, future teachers. This number may introduce a bias in perceptions and undermine the representativeness of the other four groups of stakeholders. A more balanced sample should be created in future studies.

Third, we used online social media platforms for PE teachers. The use of such platforms challenges the researcher's ability to ensure representativeness of the participants, and therefore, we cannot exclude a potential self-selection bias.

In conclusion, our study highlighted the broad expectations placed upon PE teachers, from fostering physical health and motor skills to promoting well-being and personal growth. These expectations stress the need to equip PE teachers with appropriate tools to address the full range of the student's needs, especially in light of the increasing mental health and physical inactivity challenges faced by today's children and youth. Additional studies should further explore what is expected of the PE teacher to do in class in order to maintain, and even strengthen, its relevancy at the current time.

## Data Availability

The raw data supporting the conclusions of this article will be made available by the authors, without undue reservation.

## Appendix 1

Full list of the PE teachers’ tasks that were presented to the stakeholders.

**Table:**

1	Encouraging a healthy and active lifestyle
2	Promoting engagement in physical activity
3	Teaching a range of sport disciplines
4	Developing sport skills
5	Enhancing children's self-confidence
6	Encouraging involvement in sport
7	Introducing a variety of physical activities
8	Enhancing motor skills
9	Instilling social and cultural values
10	Shaping children according to sport-related values (e.g., competitiveness or dedication)
11	Educating through physical empowerment
12	Supporting children's mental health
13	Fostering self-esteem
14	Promoting health and well-being
15	Supporting developmental processes and motor skill acquisition
16	Creating opportunities for self-expression and creativity
17	Improving children's body composition
18	Delivering knowledge and deepening understanding
19	Fostering the development of physical literacy
20	Addressing individual needs
